# Global and regional trends of atmospheric sulfur

**DOI:** 10.1038/s41598-018-37304-0

**Published:** 2019-01-30

**Authors:** Wenche Aas, Augustin Mortier, Van Bowersox, Ribu Cherian, Greg Faluvegi, Hilde Fagerli, Jenny Hand, Zbigniew Klimont, Corinne Galy-Lacaux, Christopher M. B. Lehmann, Cathrine Lund Myhre, Gunnar Myhre, Dirk Olivié, Keiichi Sato, Johannes Quaas, P. S. P. Rao, Michael Schulz, Drew Shindell, Ragnhild B. Skeie, Ariel Stein, Toshihiko Takemura, Svetlana Tsyro, Robert Vet, Xiaobin Xu

**Affiliations:** 1 NILU -Norwegian Institute for Air Research, Kjeller, Norway; 2 Norwegian Meteorological Institute, Oslo, Norway; 3 QA/SAC Americas, WMO/GAW, Champaign, IL USA; 4 Institute for Meteorology, Universität Leipzig, Leipzig, Germany; 5 NASA Goddard Institute for Space Studies and Center for Climate Systems Research, Columbia University, New York, USA; 6 Cooperative Institute for Research in the Atmosphere, Colorado State University, Fort Collins, CO USA; 7 International Institute for Applied Systems Analysis (IIASA), Laxenburg, Austria; 8 Laboratoire d’Aérologie, Université de Toulouse, CNRS, UPS, Toulouse, France; 9 National Atmospheric Deposition Program (NADP), Champaign, IL USA; 10 Center for International Climate and Environmental Research – Oslo (CICERO), Oslo, Norway; 11 Asia Center for Air Pollution Research (ACAP), Niigata, Japan; 12 Indian Institute of Tropical Meteorology, Pune, India; 13 Nicholas School of the Environment, Duke University, Durham, NC USA; 14 Air Resources Laboratory, NOAA, MD USA; 15 Research Institute for Applied Mechanics, Kyushu University, Fukuoka, Japan; 16 Environment and Climate Change Canada, Toronto, Canada; 17 Chinese Academy of Meteorological Sciences, Key Laboratory for Atmospheric Chemistry, China Meteorological Administration, Beijing, China

## Abstract

The profound changes in global SO_2_ emissions over the last decades have affected atmospheric composition on a regional and global scale with large impact on air quality, atmospheric deposition and the radiative forcing of sulfate aerosols. Reproduction of historical atmospheric pollution levels based on global aerosol models and emission changes is crucial to prove that such models are able to predict future scenarios. Here, we analyze consistency of trends in observations of sulfur components in air and precipitation from major regional networks and estimates from six different global aerosol models from 1990 until 2015. There are large interregional differences in the sulfur trends consistently captured by the models and observations, especially for North America and Europe. Europe had the largest reductions in sulfur emissions in the first part of the period while the highest reduction came later in North America and East Asia. The uncertainties in both the emissions and the representativity of the observations are larger in Asia. However, emissions from East Asia clearly increased from 2000 to 2005 followed by a decrease, while in India a steady increase over the whole period has been observed and modelled. The agreement between a bottom-up approach, which uses emissions and process-based chemical transport models, with independent observations gives an improved confidence in the understanding of the atmospheric sulfur budget.

## Introduction

There have been large changes in the global and regional SO_2_ emissions over the last decades. After a steady increase in emissions of SO_2_ since the beginning of the twentieth century^1^, the growing awareness of the negative effects of air pollution on environment and human health, gave rise to international and national legislation on emission-reductions^2–4^. European and North American SO_2_ emissions were reduced by 70–80% since 1990^2,3,5^, with the largest emission reductions in North America occurring in the last part of the period^1,3,6–8^, while in Europe the reductions were largest in the first part of the period^1,2,5,9–12^. There were also substantial reductions earlier, from 1980–1990^1^. These large regional SO_2_ emission reductions resulted in a global decrease from around 1980 until 2000^1^, after which the global emissions increased due to a sharp rise in the Chinese emissions up to around 2006, followed by a declining global trend, mainly due to stricter emission controls in China^13–20^ and trends in Europe and North America^1,4,12,14^. Not all countries have implemented effective emission controls. In India, the emissions continue to increase^1,21,22^, and India is now the world’s second largest SO_2_ emitting country after China^23^.

The large changes in SO_2_ emissions have also influenced the radiative forcing of aerosols. Sulfate aerosols have an impact on climate directly by scattering solar radiation and thus cooling the earth’s surface, and also an indirect effect on the formation of clouds and precipitation^24^. The global mean radiative forcing, due to aerosol changes over the 1990–2015 period, increased by about +0.1 W m^−2^, but the uncertainty is large^25^. The main reason for the increased positive radiative forcing of aerosols over this period is the substantial reduction of global mean SO_2_ emissions coupled with higher black carbon emissions^25^. Furthermore, the reductions of SO_2_ emissions over Europe are simulated to exert a local radiative forcing of 3–4 W m^−2^ for the same period^25^.

The trends calculated by the global aerosol models have seldom been compared to trends in observations. Trend assessments have mostly been done on regional and national observations^6–8,11,26,27^, though there are also studies which combine modeled and observed trends^9,10,28–30^. Global or hemispheric assessments have on the other hand, been done for short time periods or selected years only^31–35^, or on somewhat limited dataset^36^ and few models^19^. In this study, we have compiled monthly average mean concentrations of SO_2_, sulfate in aerosols and wet deposition of sulfate from major regional networks from 1980 onward until 2015 when available (See Fig. S1 in Supplementary Material (SM)). These trends have been compared to trends estimated by different global models for the period 1990–2015.

We address the question whether global climate models are able to reproduce the recent observed changes in the regional amplitudes of atmospheric sulfur and its inhomogeneous spatial distribution. Reproduction of historical atmospheric pollution levels is crucial to demonstrate that such models are capable to predict the impact of air pollution on climate and air quality in future scenarios. In turn, the consistency allows to clearly attribute the observed concentration changes to the emission changes. This is particularly important since a central objective of the long-term regional monitoring programmes is to document changes in atmospheric composition and test the effectiveness of environmental policies. This work may demonstrate the usefulness of strengthening the international cooperation among regional measurement networks.

## Results and Discussions

### How have the changes in SO_2_ emissions affected average regional sulfate concentrations?

**Figure 1:**
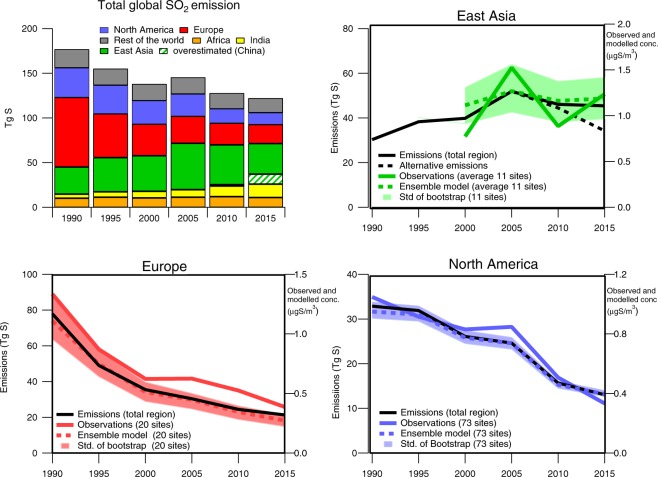
Ensemble modeled and observed trends of sulfate in aerosols over the period 1990–2015 compared to the trend in emissions over the same period. The upper left panel includes a striped green part indicating possible overestimated emissions in China, and the dotted black line in the East Asia panel shows an alternative emission trend adjusted from more recent inventories^18,20^. The time series show the annual values for years given. The uncertainty is illustrated using the standard deviation of the bootstrap trend for each region.

The regional contributions to the global trends in sulfur emissions from 1990 to 2015 are illustrated with five year intervals in Fig. 1. The regional evolution of emissions are compared to annual average observed and modeled sulfate concentrations at the sites with measurements of sulfate in aerosols since 1990 (North America and Europe) or 2000 (East Asia). The model results are given as an ensemble mean from six global models. A complete statistical trend analysis of all the measured and modeled data of sulfate in aerosols, for several (sub-)periods is given in Table 1. The observations, emissions, and the model results show a consistent and substantial change in the regional sulfur budgets.

**Table 1:** Average absolute and per cent trends in sulfate in aerosols.

Time period	Region	Nr. of stations	Average annual trend (STD) %/year		Absolute annual trend (STD) μgS/m^3^ year	
			Obs	Ensemble	Obs	Ensemble
1980–1990	Europe	16	−2.56 (3.10)		−0.048 (0.094)	
1990–2000	Europe	41	−5.16 (2.11)	−5.23 (1.17)	−0.073 (0.052)	−0.070 (0.051)
1990–2000	North America	101	−2.08 (1.44)	−1.94 (0.43)	−0.024 (0.025)	−0.021 (0.014)
1990–2015	Europe	33	−2.93 (0.69)	−3.10 (0.48)	−0.031 (0.015)	−0.036 (0.023)
1990–2015	North America	124	−2.14 (0.67)	−2.19 (0.29)	−0.026 (0.024)	−0.024 (0.018)
2000–2010	East Asia	13	0.44 (4.25)	0.42 (1.15)	0.003 (0.034)	0.006 (0.013)
2000–2010	Europe	43	−2.86 (2.20)	−3.53 (1.06)	−0.029 (0.041)	−0.027 (0.017)
2000–2010	North America	227	−3.03 (1.72)	−3.22 (0.77)	−0.029 (0.029)	−0.027 (0.020)
2000–2015	East Asia	13	**2.68** (**9.41**)	**0.02** (**0.91**)	0.003 (0.037)	0.001(0.009)
2000–2015	Europe	36	−2.67 (2.03)	−3.26 (0.85)	−0.025 (0.028)	−0.025 (0.015)
2000–2015	North America	218	−3.15 (1.30)	−3.18 (0.66)	−0.028 (0.029)	−0.024 (0.019)

Data in bold indicate trends not consistent with the observations within the standard deviation (STD).

Globally the SO_2_ emissions were reduced by 55 TgS (31%) from 1990 to 2015. Individual regions have had different contributions to the global emission budget throughout this period (see Fig. 1) as also documented in other studies^1,14,21,25,37^. The largest decrease in global SO_2_ emissions occurred in the first decade, from 1990–2000 and was mainly due to a large reductions in Europe (−42 TgS/−54%). There was a smaller decrease in North America (−7 TgS/−21%) during this time, and an increase in East Asia (+10 TgS/32%). In comparison, in the following period 2000–2015, emissions in Europe and the US decreased by a similar total amount (−14 and −13 TgS) or in relative terms, respectively by −40% and −50%. In Eastern Asia, there was an increase of the emissions up to 2005 by more than +20 TgS (70%), while in the last ten years from 2005 to 2015 there has been a reduction, we have used emission inventories with a decrease of −6 TgS (−13%). For the whole 25 year period from 1990 to 2015, India’s emissions increased from 4.5 to 15 TgS, while in Africa only small changes occurred, +1 TgS (8%).

The emission inventories in China, which is the main contributor to the SO_2_ emissions in East Asia, have been extensively studied the last years^13,15–18,20,38^, and the most recent estimates indicate that the negative trends in the inventory used in our study is most likely underestimated. Zheng *et al*.^20^ estimate SO_2_ reduction in China of about 62% in the period 2010–2017, with the largest decrease after 2013 when the China’s Clean Air Action was implemented. This rapid decline in the recent years appears to be confirmed with satellite observations^20,23,39^. The main reason for these differences is that achieved effectiveness of policies implemented following China’s Clean Air Action plans was not anticipated in earlier inventories. To illustrate the difference between the emissions used by the models in this study and the most recent estimates for the last ten year period in East Asia, the new inventories are included in Fig. 1, showing a decrease of −18 TgS (−34%) between 2005–2015^18,20^; (shown as ‘Alternative emissions’).

The aerosol sulfate trends in observations and ensemble model results at the measurement sites with long term trends compare well with the trends of SO_2_ emissions for all regions, especially North America and Europe (See Fig. 1). The ensemble model results and observations give comparable annual reductions of sulfate in aerosols by around 5.2%/y and 2.0%/y in Europe and North America, respectively, for the period 1990–2000 (Table 1). For the 2000–2015 period, the ensemble model and observations agree with 3.1%/y reduction in North America, while in Europe the ensemble model mean shows a higher relative reduction of 3.3%/y, compared to 2.7%/y in the observations, although the absolute changes are similar (−0.025 μgS/y, see Table 1), though the modelled and observed trends are comparable within the uncertainties for both relative and absolute changes per year.

The temporal development in East Asia was different with an increase in SO_2_ emissions up to 2005, and a decline thereafter^1,14,17,20,40^. The observations from this region do not capture the complete period, starting only in 2000, with relatively few sites. However they indicate the same tendency for an increase from 2000 to 2005 followed by a decline from 2005 to 2010. For the 2000–2010 period, both the ensemble model mean and the observations show a small average increase of 0.4%/y, although with quite high uncertainty, Table 1. There is an observed increase from 2010 to 2015, though it should be emphasized that the variability between the sites is high, and when considering that the emissions most probably have decreased more in the last five-year period, the increase in the observed sulfate concentrations seems to be non-representative. One should note that none of the sites with aerosols measurements used in this study are located in China. The large positive trend is mainly caused by two sites in Indonesia with 20–30% increases, which were not captured by the models. Whether this was due to local influence or long-range transport is difficult to say. It could be due to influences from volcanic activities in the region, but there does not seem to be an increase in eruptions the latter years^41^ nor do we see the same signal for the measured SO_2_.

### Comparability between sulfur trends in gaseous phase, aerosols and wet deposition

**Figure 2:**
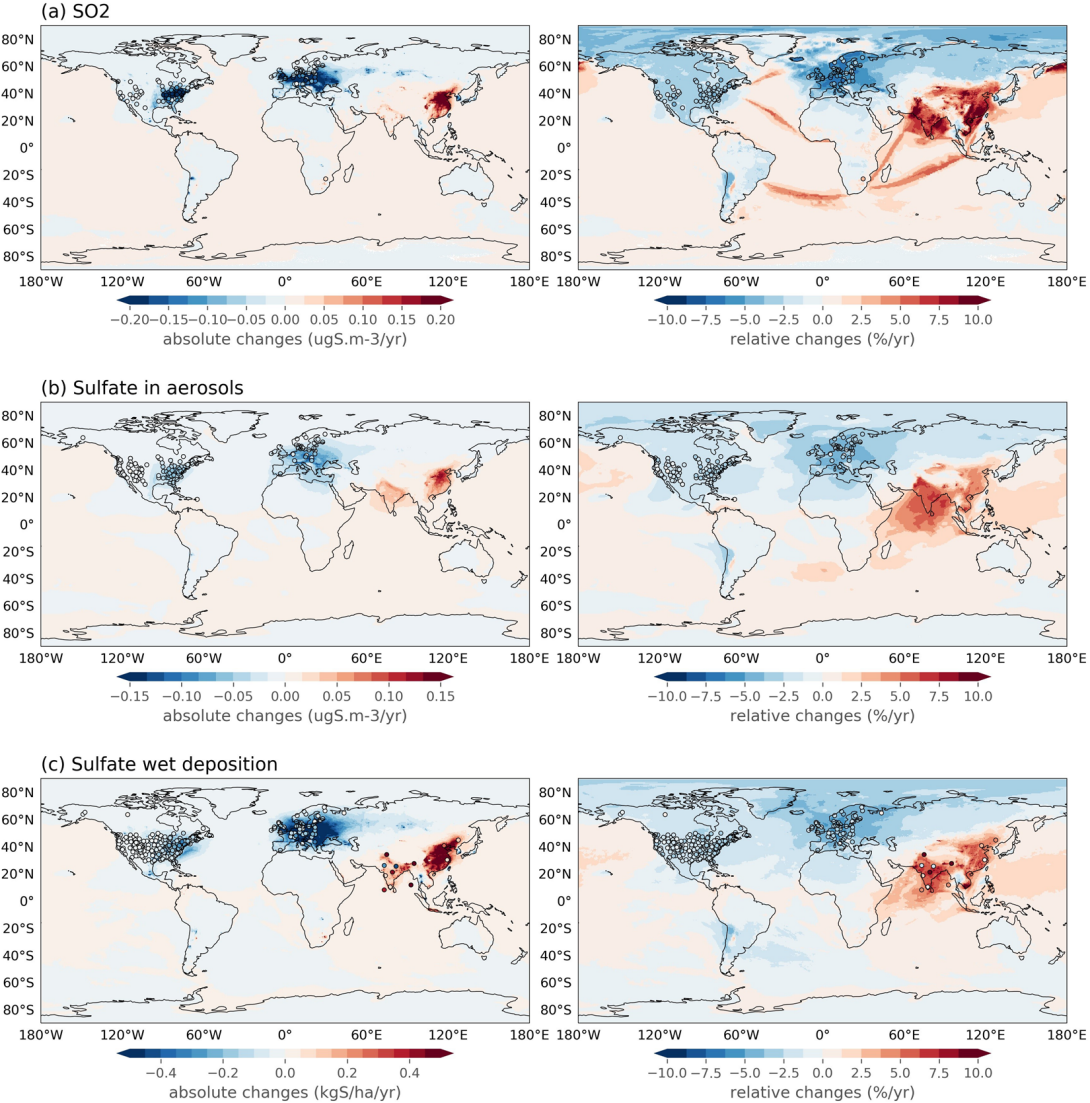
Absolute (left) and relative (right) trends of air concentrations and wet deposition calculated by the EMEP MSC-W model with observed trends superimposed (open circles), of SO_2_ (**a**) and sulfate in aerosol (**b**) and wet deposition of sulfate (**c**) over the 1990–2015 period.

The trends of SO_2_, sulfate in aerosols and sulfate in precipitation were compared using the EMEP MSC-W model result and the global observation set. It is important to note that the EMEP MSC-W model results are close to the ensemble mean for aerosol sulfate, as discussed in the next section. In addition to the measurement sites that provided long term observations of sulfate in aerosols (discussed above), observations of sulfate in precipitation were available from sites in Africa, India, and additional Chinese sites (Table 2). For SO_2_, there was additional observational data from Africa (Table 3). Figure 2 shows the modeled global distribution of absolute and relative trends of concentrations and deposition. Comparing the maps with relative and absolute changes, the former visualize the trends better in areas where the concentrations or depositions are relatively low. This is especially visible in India where the relative changes are particularly large while the absolute changes are not, further note considerable positive trends from ship traffic, which has increased during the last decades^1,14^.

**Table 2:** Average absolute and per cent trends in wet deposition of sulfate.

Time period	Region	Nr. of stations	Average annual trend (STD) %/year		Absolute annual trend (STD) kgS/ha year	
			Obs	EMEP MSC-W	Obs	EMEP MSC-W
1980–1990	Europe	23	−2.37 (2.33)		−0.30 (0.36)	
1980–1990	India	10	13.7 (25.6)		0.00 (0.18)	
1980–1990	North America	78	−1.80 (4.09)		−0.06 (0.18)	
1990–2000	East Asia (China)	3	**−1.27 (2.67)**	**5.07 (0.36)**	**−0.16 (0.32)**	**0.68 (0.14)**
1990–2000	Europe	60	−4.02 (4.44)	−6.41 (2.17)	−0.29 (0.29)	−0.91 (0.71)
1990–2000	India	10	**22.6 (16.0)**	**4.31 (1.53)**	0.42 (0.37)	0.18 (0.14)
1990–2000	North America	186	−1.84 (2.33)	−1.93 (0.48)	−0.10 (0.13)	−0.14 (0.11)
1990–2015	East Asia (China)	3	**1.15 (0.74)**	**2.84 (0.23)**	**0.08 (0.05)**	**0.44 (0.11)**
1990–2015	Europe	55	−3.03 (0.93)	−3.74 (0.49)	−0.16 (0.12)	−0.40 (0.26)
1990–2015	North America	189	−2.17 (0.65)	−2.41 (0.26)	−0.11 (0.10)	−0.19 (0.14)
2000–2010	Africa (Lamto)	1	**4.15**	**2.69**	0	0
2000–2010	East Asia	30	0.49 (4.14)	0.85 (2.01)	0.04 (0.37)	0.07 (0.28)
2000–2010	Europe	73	−3.85 (2.83)	−4.36 (1.22)	−0.16 (0.13)	−0.26 (0.19)
2000–2010	India	10	2.27 (8.48)	5.64 (2.21)	−0.07 (0.37)	0.29 (0.17)
2000–2010	North America	226	−2.30 (2.74)	−3.79 (0.68)	−0.12 (0.13)	−0.25 (0.19)
2000–2015	East Asia	30	−0.98 (2.48)	0.16 (1.33)	−0.12 (0.24)	0.05 (0.23)
2000–2015	Europe	67	−3.40 (1.37)	−3.94 (0.96)	−0.12 (0.10)	−0.23 (0.16)
2000–2015	North America	215	−2.78 (2.02)	−3.75 (0.65)	−0.13 (0.13)	−0.23 (0.18)

Data in bold indicate trends not consistent with the observations within the standard deviation (STD).

**Table 3:** Average absolute and per cent trends in SO_2_.

Time period	Region	Nr. of stations	Average annual trend (STD) %/year		Absolute annual trend (STD) μgS/m^3^ year.	
			Obs	EMEP MSC-W	Obs	EMEP MSC-W
1980–1990	Europe	20	−5.03 (2.04)		−0.211 (0.168)	
1990–2000	Europe	43	−7.56 (1.81)	−8.54 (1.40)	−0.220 (0.275)	−0.318 (0.316)
1990–2000	North America	53	−3.27 (1.69)	−2.63 (0.30)	−0.125 (0.115)	−0.065 (0.037)
1990–2015	Europe	40	−4.43 (0.88)	−4.63 (0.43)	−0.084 (0.085)	−0.131 (0.132)
1990–2015	North America	71	−3.14 (0.75)	−2.83 (0.30)	−0.116 (0.109)	−0.066 (0.046)
2000–2010	Africa	8	**17.6** (**13.9**)	**1.86** (**1.19**)	0.144 (0.121)	0.012 (0.025)
2000–2010	East Asia	19	5.84 (9.40)	0.35 (2.22)	0.038 (0.119)	0.001 (0.025)
2000–2010	Europe	51	−4.23 (3.17)	−5.31 (1.61)	−0.046 (0.054)	−0.064 (0.055)
2000–2010	North America	78	−4.55 (1.68)	−4.44 (0.95)	−0.119 (0.113)	−0.080 (0.060)
2000–2015	Africa	8	**4.97** (**2.70)**	**1.11** (**0.63)**	0.062 (0.068)	0.008 (0.011)
2000–2015	East Asia	19	−0.14 (5.32)	−0.41 (0.92)	−0.055 (0.186)	−0.001 (0.031)
2000–2015	Europe	47	−3.89 (2.16)	−4.86 (1.31)	−0.036 (0.036)	−0.054 (0.046)
2000–2015	North America	77	−4.69 (1.35)	−4.40 (0.93)	−0.130 (0.123)	−0.069 (0.051)

Data in bold indicate trends not consistent with the observations within the standard deviation (STD).

Concentrations of the primary (directly) emitted compound, SO_2_, show greater decreases than the secondary (formed via atmospheric chemistry) sulfate in aerosols and in precipitation (Tables 1–3 and Figs S3–S5 in the SM). This non-linear relationship is seen in both model results and observations in North America and in Europe. This may partly be explained by the increasing oxidation capacity of the atmosphere during these twenty years^7,42–44^. In the early 1990’s, SO_2_ emissions were still high, and the oxidation of SO_2_ to sulfate was limited to some extent by the availability of the oxidants H_2_O_2_, OH and O_3_. As the emissions decreased, more oxidants became available, and SO_2_ was more efficiently oxidized to sulfate. Furthermore, a very important factor is that the decrease in SO_2_ emissions (and only slightly decreasing ammonia emissions) has led to less acidic cloud droplets, which increased the oxidation rate of SO_2_ to sulfate via the ozone pathway^9,30,45^. In addition, less acidity in the environment probably leads to more efficient dry deposition of SO_2_^42,43,46^, which would also contribute to a larger reduction in SO_2_ concentrations with respect to sulfate. The trends in wet deposition of sulfur are lesser than the trends in SO_2_, but larger than those of sulfate in aerosols, because both SO_2_ and sulfate are efficiently scavenged by rain, and the wet deposition trend therefore represents a mix of SO_2_ and sulfate in aerosols.

The somewhat puzzling increase in the observed sulfate concentration in aerosol seen in East Asia for 2010 to 2015 is not observed for SO_2_ nor in sulfate in precipitation (Tables 1–3 and Figs S3–S5 in the SM). These show a steady decline from 2005–2015 and are more in line to the more recent inventories with a larger decrease in SO_2_ emissions since 2010 period than the emissions used in the model calculations.

### Comparability between the global models

**Figure 3:**
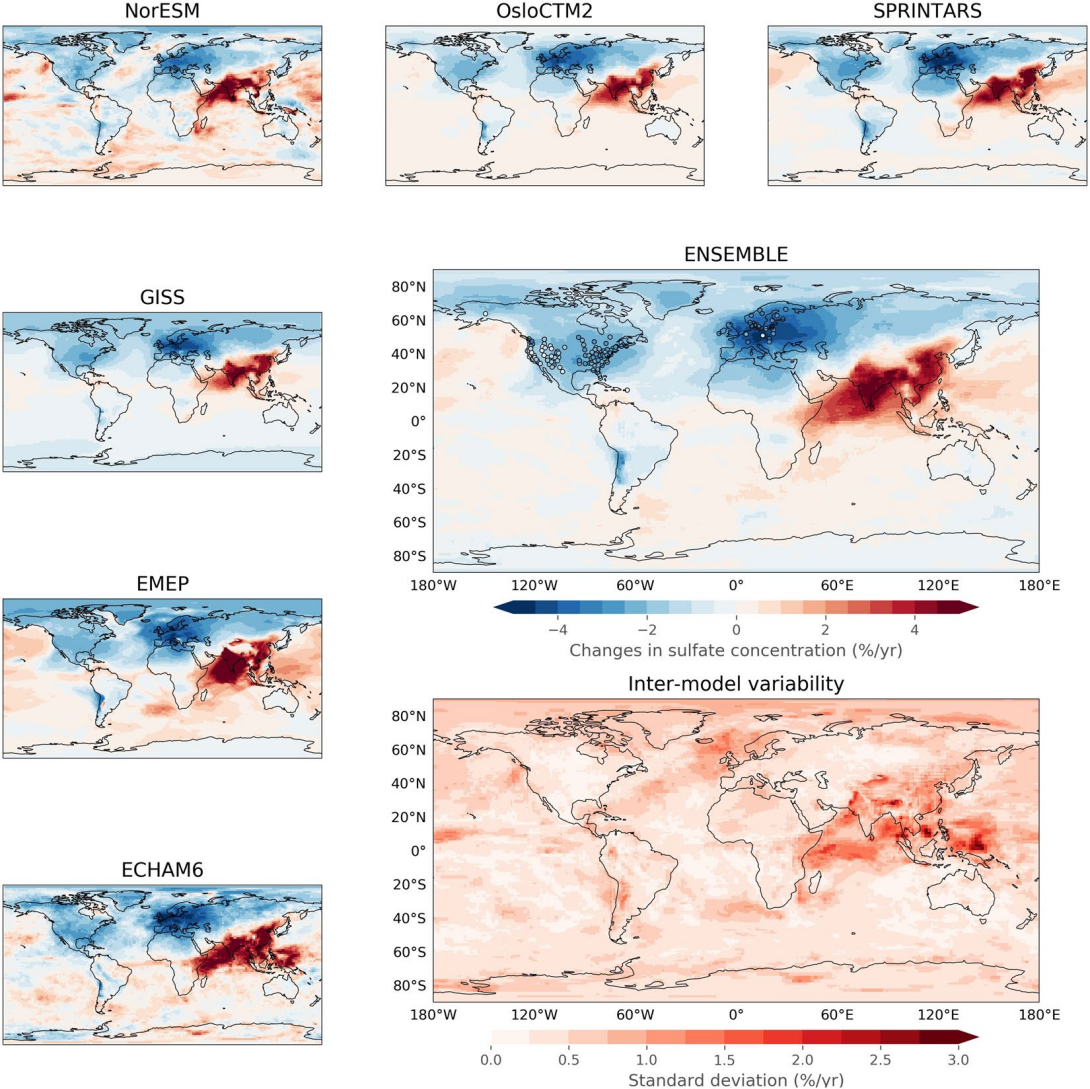
Relative trends in sulfate concentrations in aerosol for 1990–2015, calculated by the individual and ensemble models, with the observed trends superimposed (open circles). The differences between the models are shown in the inter-model variability map.

The spatial variability in the aerosol sulfate trends calculated by the individual models for the whole period 1990–2015 is shown in Fig. 3, including also the ensemble model mean and the standard deviation between the models. The general pattern is quite well represented by all the models. Quantitatively, the models show very similar trends in North America and Europe, while in East Asia the spatial differences are larger. Notably some of the largest differences are found over the Himalayas, which is also the boundary between areas with upward and downward trends. Some of the differences between the models might be explained by how fast the models oxidize SO_2_ and the lifetime of sulfate in aerosols^47^.

**Figure 4:**
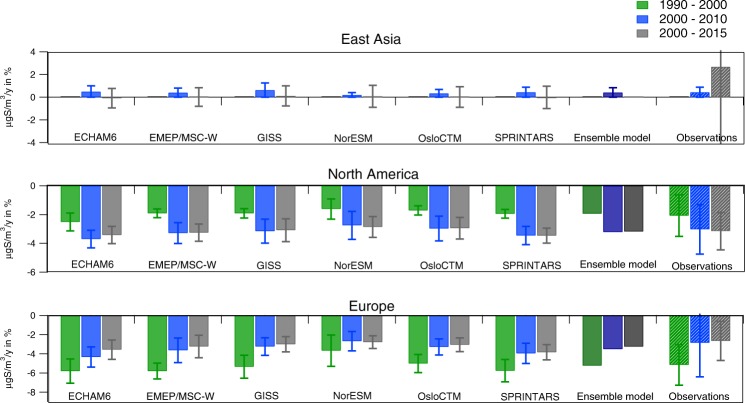
Comparison of the relative trends in sulfate concentrations in aerosol calculated by the different global models at sites with observed trends in the selected periods. The error bars indicate the standard deviation between the sites.

When comparing the average relative trends of sulfate based on the observations and the models for different periods in Fig. 4 (the statistical information is found in Table S1 and S2 in the SM), the different models give similar trends as the observations, but there are systematic differences between them. NorESM gives the lowest relative reductions, while Sprintars and ECHAM6 generally give the highest reductions. The differences between the models are larger for Europe compared to North America, maybe due to fewer sites in Europe representing the region. For the modeled average relative trends for the regions defining the largest emission areas in North America and Europe, the systematic differences in the relative trends between the models are smaller (Fig. S6 in SM). When comparing the average concentrations for the regions, there is a large spread between the models, but the differences depend on how the regions are defined since the models show somewhat different spatial patterns.

### Spatial representativeness of the regional trends

The number of sites needed to quantify and validate emission changes depends on both the spatial and temporal variability of the trends. This is true for both models and observations, but the variability in the measurements is higher (see box plot in Fig. S5 in SM), thus more difficult to detect significant trends. For example, in Europe, the models seem to simulate somewhat higher trend from 2000 onwards compared to the observations (see Fig. 4, Tables 1–3), although the differences are within the standard deviation. These variations can partly be explained by some sites with quite low concentration levels and high relative changes (though not necessarily significant trends), influencing the average relative trends. The models compare well with the trends at those sites with large significant reductions, which can be seen for the trends for the 25th percentile of the sites with the highest rate, with a change of −4.1%/y and −3.8%/y for the period from 2000 to 2015 for the ensemble model mean and observations for sulfate in aerosols, respectively.

In East Asia there are positive and negative trends depending on periods and components, while in India and Africa there are positive trends, but the variability in the observations are high and there are relatively few sites for all these three regions. The sites do not necessarily represent the whole regions. In Africa, SO_2_ increases at a rate of 17 ± 14%/y from 2000 to 2010, but the increase occurred only in South Africa due to the influence of increased coal burning^48^. At West- and Central African sites the concentrations are low and no significant trends are apparent, as reported earlier by Adon *et al*.^48^. In China, there are three sites with precipitation measurements from 1990 on but, except for one site, these do not show the increase indicated by the models and by the emission trends for the 1990–2000 period. However, by extending the period to 2005, the sites indicate an increase, illustrating the sensitivity of the results to the choice of time period. In India the sites are quite well spread over the country and there is a clear increase in the observed wet deposition in all the periods, which is also seen in the emissions and models. However, the magnitude of the trend is uncertain due to quite large variabilities and scattering in the time series.

A bootstrap approach has been used to assess how well the number of sites available represents each region. This is a possible method of assessing the representativeness of the trends, which, however, has its deficiencies. For example, changes in the absolute concentrations in North America, where most areas are remote, one is much more likely to pick a point in low rather than high concentration areas, while a large fraction of the observational sites are located closer to the higher emission and concentration regions. Thus the average observed concentrations are higher than in the bootstrap analysis results (Fig. S3 in SM). However, the standard deviation around the bootstrap mean is an indicator of the uncertainty caused by the geographical location of the given number of sites within each region. Figure 1 shows a high standard deviation of the sulfate concentrations in East Asia due to the relatively small number of sites and the inhomogeneous emission changes in this region.

For normalized trends (Fig. S4 in SM), the trends in the bootstrap averages are consistent with the modeled trends in North America for all the species, while in Europe the modeled trends at the sites are larger than the bootstrap average. This suggests that the site density in North America is representative for trend analysis for the whole region while in Europe the number of sites is apparently too low for a representative estimate. The normalized trends in emissions are most consistent with the trends in sulfate in aerosols, indicating that this is a relevant parameter for compliance monitoring. In East Asia, the normalized modeled trends from 1990–2005 show smaller increase than the bootstrap average, especially for SO_2_, which indicates that the sites are not located where the largest changes in the region have taken place, nevertheless the observations show larger changes for SO_2_ and aerosol sulfate, though as discussed there are quite large variability between the sites in addition to the uncertainties in the emission inventory in this region.

## Conclusions

The results give confidence in the global aerosol models’ abilities to calculate historical sulfur trends and, thus, their scenario analyses of their future impact on climate and air quality. The fact that trends of emitted sulfur, sulfur in the aerosol phase after chemical transformation in the atmosphere, and sulfur in precipitation after wet removal agree between the observations and the model further implies that the relevant processes are realistically represented in the models.

However, one needs to be cautious when drawing conclusions from trends in regions with poor measurement site coverage, like India and China, and to some degree Africa, Australia and South America. There is a strong need for more sulfur observations in air, aerosols and precipitation to enable more homogeneous global coverage. Still, the work here reveals consistent trends on regional and global scales between the two independent methods. The good agreement also enhances confidence in the emission inventories in North America and in Europe and, in turn, confirms that the concentration changes are attributable to SO_2_ emission changes. However, the bottom up emission inventory in East Asia used in this study has not captured the most likely large decrease in emissions in China after 2013. Further studies in this region require in depth comparison between updated emission inventories, atmospheric transport models, *in situ*- as well as satellite observations to better describe the significant changes in sulfur the last ten years for this region. The large ongoing increase in sulfur emissions in India also needs further investigations and should be more closely monitored.

This work has illustrated the strength of close co-operation between the regional networks to assemble a harmonized comparable global dataset. Future work on global trend analysis combining models and observations should include other species, in particular nitrogen compounds, organic and absorbing carbon are of high interest.

## Methods

### Global dataset of sulfur observations

Measurement data were collected from different regional and global networks, in total 365 sites. A map showing the sites is found in Supplementary Fig. S1. More information on the networks, methods and access to original data can be found in the references and links given in Table 4.

**Table 4:** List of networks contributing with sites and data. The original data can be accessed from the given web pages.

Acronym and references	Network	Region
CAPMoN^31,51^	Canadian Air and Precipitation Monitoring Network Including the New Brunswick Precipitation Network (NBPN), https://open.canada.ca/en/open-data	Canada
CASTNET^7,51^	Clean Air Status and Trends Network, https://www.epa.gov/castnet	US
EANET^31,51^	Acid Deposition Network in East Asia, http://www.eanet.asia/	East Asia
EMEP^10,11,31,51^	The European Monitoring and Evaluation Programme, http://ebas.nilu.no/	Europe
INDAAF^31,51^	International Network to study Deposition and Atmospheric chemistry in Africa, https://indaaf.obs-mip.fr/	Africa
IMPROVE^8,38^	Interagency Monitoring of Protected Visual Environments, http://vista.cira.colostate.edu/improve/	US
NADP^6,31^	National Atmospheric Deposition Program. Including data from MAP3S-AIRMoN (Atmospheric Integrated Research Monitoring Network), https://nadp.slh.wisc.edu	US
GAW - China	Global Atmosphere Watch, regional sites in China	China
GAW - India^26,31^	Global Atmosphere Watch, regional sites in India	India
WDCPC^31^	WMO/GAW World Data Centre for Precipitation Chemistry, http://www.wdcpc.org/	Global

Precipitation and wet deposition sulfate measurements are mainly from wet-only samplers or bulk samplers if proven comparable to wet-only and from similar sites as was described elsewhere^31^. The sulfate aerosol measurements are done using the aerosol filters, either PM_10_ or no size cut off like the filterpack method, except for the IMPROVE network, which uses PM_2.5_ filters^8^. For SO_2_, the filterpack method is commonly used in North America and Europe, while in Africa passive samplers dominate. In East Asia, both these methods in addition to continuous monitors are used. The sampling frequency is mostly daily and sometimes weekly, except for African data for which precipitation is sampled by event and SO_2_ with monthly passive samplers. Wet deposition and volume-weighted precipitation data are based on the standard rain gauge depth if that is measured in parallel. At other sites without rain gauge, the sample precipitation amount is used. Monthly means are calculated for months with 70% or better data coverage. Urban sites are not included, and sites where the surroundings have changed considerably over the years have been excluded when known. The sites have been screened to be regionally representative with data of satisfactory quality.

### Global modelling and emission estimates

The global models involved in the present study are described in Table 5. The model setup to simulate the sulfur changes varies between the models; from any fixed meteorology, to that of one particular meteorological year, to just fixed sea surface temperatures. All models simulated and diagnosed sulfate in aerosol, while only one model (EMEP/MSC-W) simulated SO_2_ and wet deposition of sulfate for this study. In addition to sulfate, all the models simulated black carbon (BC) and primary organic aerosols (POA), and several also included secondary organic aerosols, nitrate, sea salt and dust. Details are found in Myhre *et al*.^25^ and in the references in Table 5. All models use identical anthropogenic emission data from the EU project ECLIPSE for the 1990 to 2015 period; several updates and improvements compared to earlier emission data sets were included in this inventory^21,49^. A direct link to the respective dataset is found here: http://www.iiasa.ac.at/web/home/research/researchPrograms/air/ECLIPSEv5a.html.

**Table 5:** Model descriptions.

Models and references	Resolution	Fixed-met or fixed-SST
ECHAM6-HAM2^52,53^	T63 1.8° × 1.8° L31	Climatological monthly varying fixed-SST and sea ice extent averaged for the period 1979 to 2008.
EMEP/MSC-W^54^	0.5° × 0.5° L20	2010 meteorology, 3 hourly ECMWF based
GISS^55,56^	2.0° × 2.5° L40	2000 climatological monthly varying fixed-SSTs and sea-ice
NorESM1^57–59^	1.9° × 2.5° L26	Climatological monthly varying fixed SSTs and sea ice extent over the 1990–2013 period
OsloCTM2^60,61^	T42 2.8° × 2.8° L60	2010 meteorology, 3 hourly ECMWF based
SPRINTARS^62,63^	1.125° × 1.125° L56	Climatological monthly varying fixed SSTs and sea ice extent over the 1988–1992 period

Natural emissions from i.e. volcanos are included but do vary somewhat between the models. See the references in Table 5 for details.

### Statistical calculations

For each of the periods considered, the sites were selected based on the criteria that number of available years should be at least 75% of the total number of years in the period. The statistics are done from both yearly and seasonal averages, though only yearly are included. At least 10 measurements are required per season for being used in the statistics. The yearly averages are computed from the seasonally averages if the four seasonal averages are available (with more than 10 measurements) per year. Data from the same sites and periods have been extracted from the model output and trends have been calculated in similar way. Note that for most of the models (except NorESM and ECHAM6), there are only modelled data for each 5 year period. For the statistical analysis, the non-parametric Mann-Kendall test has been used on annual means for detecting and estimating trends; this is a common method when missing values occur and when data are not normally distributed^10,11^. In parallel, the Theil-Sen’s slope estimator has been used to quantify the magnitude of potential trends^50^. A 90% confidence interval (p-value below 0.10) was chosen as the criterion for determining the significance of the trend. The Scientific Library for Python (scipy-0.14.0) has been used for calculating the trends. The average per cent changes are calculated for all the sites, not only for those with a significant trend. The robustness in the trends in modelled data for shorter periods (10–15 years) is hampered by having only few data point, i.e. 3 points for a ten year period. The Man Kendall test allows for calculation even down to 3 points, but with pval < 0.1 it is necessary with 4 points of more to estimate significance or not.

To estimate how well the selected number of sites represents the trends in their region, a bootstrap approach was used. Using the same X number of observation sites, X arbitrary inland pixels were chosen and bootstrapped with 1000 iterations. The mean and standard deviation of these 1000 iterations were used to assess the representativeness of the monitoring networks.

## Supplementary information

Supplementary material

## Data Availability

The aggregated observational global data set with monthly mean concentrations and total wet deposition values, together with the model results at the same sites have, been associated with a specific DOI and can be downloaded from 10.21336/gen.2. The statistical results from all the individual time series are also available. *A web tool*, http://aerocom.met.no/trends/S-trends/ maps the trends in SO_2_ and sulfate in aerosol and wet deposition.
