# Author Correction: Global and regional trends of atmospheric sulfur

**DOI:** 10.1038/s41598-020-62441-w

**Published:** 2020-03-24

**Authors:** Wenche Aas, Augustin Mortier, Van Bowersox, Ribu Cherian, Greg Faluvegi, Hilde Fagerli, Jenny Hand, Zbigniew Klimont, Corinne Galy-Lacaux, Christopher M. B. Lehmann, Cathrine Lund Myhre, Gunnar Myhre, Dirk Olivié, Keiichi Sato, Johannes Quaas, P. S. P. Rao, Michael Schulz, Drew Shindell, Ragnhild B. Skeie, Ariel Stein, Toshihiko Takemura, Svetlana Tsyro, Robert Vet, Xiaobin Xu

**Affiliations:** 1 NILU -Norwegian Institute for Air Research, Kjeller, Norway; 2 Norwegian Meteorological Institute, Oslo, Norway; 3 QA/SAC Americas, WMO/GAW, Champaign, IL USA; 4 Institute for Meteorology, Universität Leipzig, Leipzig, Germany; 5 NASA Goddard Institute for Space Studies and Center for Climate Systems Research, Columbia University New York, USA; 6 Cooperative Institute for Research in the Atmosphere, Colorado State University, Fort Collins, CO USA; 7 International Institute for Applied Systems Analysis (IIASA), Laxenburg, Austria; 8 Laboratoire d’Aérologie, Université de Toulouse, CNRS, UPS, Toulouse, France; 9 National Atmospheric Deposition Program (NADP), Champaign, IL USA; 10 Center for International Climate and Environmental Research – Oslo (CICERO), Oslo, Norway; 11 Asia Center for Air Pollution Research (ACAP), Niigata, Japan; 12 Indian Institute of Tropical Meteorology, Pune, India; 13 Nicholas School of the Environment, Duke University, Durham, NC USA; 14 Air Resources Laboratory, NOAA, MD, USA; 15 Research Institute for Applied Mechanics, Kyushu University, Fukuoka, Japan; 16 Environment and Climate Change Canada, Toronto, Canada; 17 Chinese Academy of Meteorological Sciences, Key Laboratory for Atmospheric Chemistry, China Meteorological Administration, Beijing, China

Correction to: *Scientific Reports* 10.1038/s41598-018-37304-0, published online 30 January 2019

This Article contains a repeated typographical error, where the wrong unit has been used for the emissions of sulfur. In this Article, the unit “TgS” has been used instead of the correct unit “TgSOx (as SO_2_)” or “TgSO_2_” for simplification. As such, in the “Results and Discussions” section:

“Globally the SO_2_ emissions were reduced by 55 TgS (31%) from 1990 to 2015. Individual regions have had different contributions to the global emission budget throughout this period (see Fig. 1) as also documented in other studies^1,14,21,25,37^. The largest decrease in global SO_2_ emissions occurred in the first decade, from 1990–2000 and was mainly due to a large reductions in Europe (−42 TgS/−54%). There was a smaller decrease in North America (−7 TgS/−21%) during this time, and an increase in East Asia (+10 TgS/32%). In comparison, in the following period 2000–2015, emissions in Europe and the US decreased by a similar total amount (−14 and −13 TgS) or in relative terms, respectively by −40% and −50%. In Eastern Asia, there was an increase of the emissions up to 2005 by more than +20 TgS (70%), while in the last ten years from 2005 to 2015 there has been a reduction, we have used emission inventories with a decrease of −6 TgS (−13%). For the whole 25 year period from 1990 to 2015, India’s emissions increased from 4.5 to 15 TgS, while in Africa only small changes occurred, +1 TgS (8%).”

should read:

“Globally the SO_2_ emissions were reduced by 55 TgSO_2_ (31%) from 1990 to 2015. Individual regions have had different contributions to the global emission budget throughout this period (see Fig. 1) as also documented in other studies^1,14,21,25,37^. The largest decrease in global SO_2_ emissions occurred in the first decade, from 1990–2000 and was mainly due to a large reductions in Europe (−42 TgSO_2_/−54%). There was a smaller decrease in North America (−7 TgSO_2_/−21%) during this time, and an increase in East Asia (+10 TgSO_2_/32%). In comparison, in the following period 2000–2015, emissions in Europe and the US decreased by a similar total amount (−14 and −13 TgSO_2_) or in relative terms, respectively by −40% and −50%. In Eastern Asia, there was an increase of the emissions up to 2005 by more than +20 TgSO_2_ (70%), while in the last ten years from 2005 to 2015 there has been a reduction, we have used emission inventories with a decrease of −6 TgSO_2_ (−13%). For the whole 25 year period from 1990 to 2015, India’s emissions increased from 4.5 to 15 TgSO_2_, while in Africa only small changes occurred, +1 TgSO_2_ (8%).”

Additionally,

“To illustrate the difference between the emissions used by the models in this study and the most recent estimates for the last ten year period in East Asia, the new inventories are included in Fig. 1, showing a decrease of −18 TgS (−34%) between 2005–2015^18,20^; (shown as ‘Alternative emissions’).”

should read:

“To illustrate the difference between the emissions used by the models in this study and the most recent estimates for the last ten year period in East Asia, the new inventories are included in Fig. 1, showing a decrease of −18 TgSO_2_ (−34%) between 2005–2015^18,20^; (shown as ‘Alternative emissions’).”

Finally, the incorrect unit is also displayed in Figure 1 and Figure S3. The correct Figures 1 and Figure S3 are displayed as Figures 1 and 2 below.

**Figure 1:**
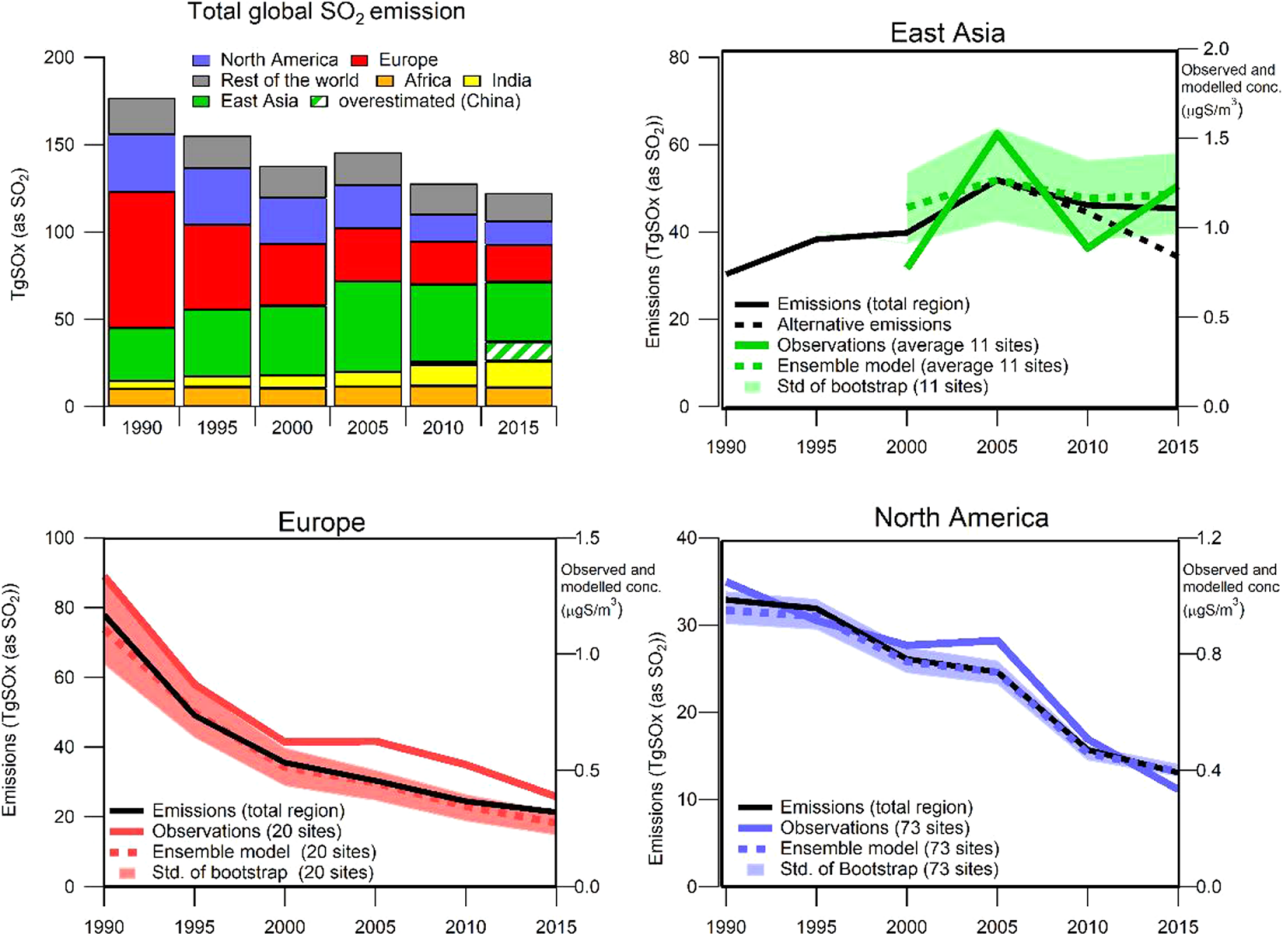
Ensemble modeled and observed trends of sulfate in aerosols over the period 1990–2015 compared to the trend in emissions over the same period. The upper left panel includes a striped green part indicating possible overestimated emissions in China, and the dotted black line in the East Asia panel shows an alternative emission trend adjusted from more recent inventories^18,20^. The time series show the annual values for years given. The uncertainty is illustrated using the standard deviation of the bootstrap trend for each region.

**Figure 2:**
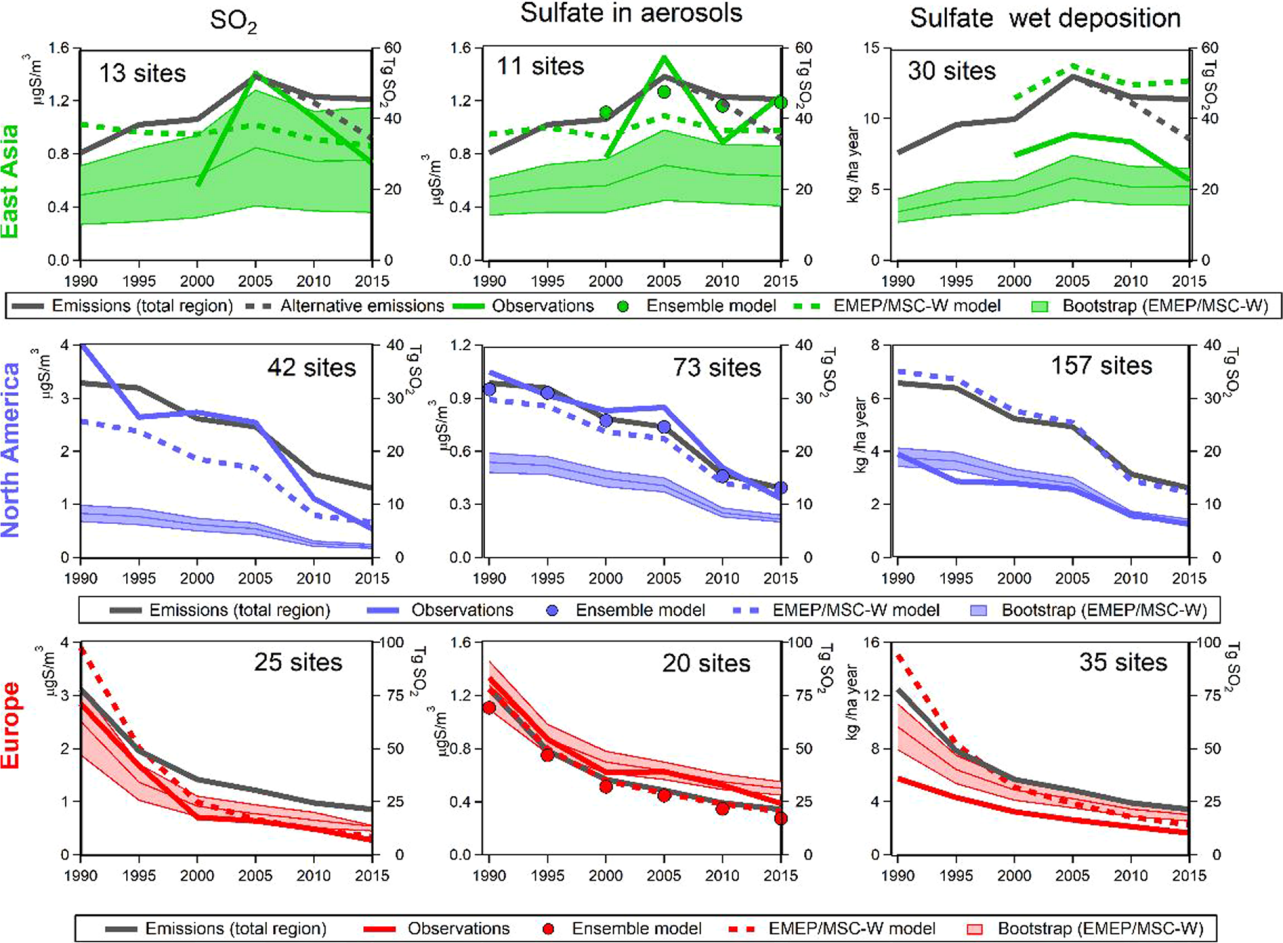
Trends in SO_2_, sulfate in aerosols and in wet deposition in East Asia, North America and Europe from observations and the EMEP/MSC-W model (and Ensemble model for sulfate in aerosols) at the selected number of sites, and the average bootstrap trends and standard deviations from 1000 iterations. Observed and modelled concentrations are given on the y-axis on the left, while emissions on the right. For East Asia an alternative emission development the last ten years are included, based on more recent inventories^7,8^.

