# Supplementary material for: Global and regional trends of atmospheric sulfur
Source: Sci Rep. 2019 Jan 30;9:953. doi: 10.1038/s41598-018-37304-0 (PMC6353995; doi:10.1038/s41598-018-37304-0)
Supplement: Supplementary file 1 — Supplementary material [file 41598_2018_37304_MOESM1_ESM.docx]

**Supplement for:**

**Global and regional trends of atmospheric sulfur**

Wenche Aas^1*^, Augustin Mortier^2*^, Van Bowersox^3^, Ribu Cherian^4^, Greg Faluvegi^5^, Hilde Fagerli^2^, Jenny Hand^6^, Zbigniew Klimont^7^, Corinne Galy-Lacaux^8^, Christopher M.B. Lehmann^9^, Cathrine Lund Myhre^1^, Gunnar Myhre^10^, Dirk Olivié^2^, Keiichi Sato^11^, Johannes Quaas^4^, P.S.P. Rao^12^, Michael Schulz^2^, Drew Shindell^13^, Ragnhild B. Skeie^10^, Ariel Stein^14^, Toshihiko Takemura^15^, Svetlana Tsyro^2^, Robert Vet^16^, Xiaobin Xu^17^

^1^NILU -Norwegian Institute for Air Research, Kjeller, Norway

^2^Norwegian Meteorological Institute, Oslo, Norway

^3^QA/SAC Americas, WMO/GAW, Champaign, IL, USA

^4^Institute for Meteorology, Universität Leipzig, Leipzig, Germany

^5^NASA Goddard Institute for Space Studies and Center for Climate Systems Research, Columbia University, New York, USA

^6^Cooperative Institute for Research in the Atmosphere, Colorado State University, Fort Collins, CO, USA

^7^International Institute for Applied Systems Analysis (IIASA), Laxenburg, Austria

^8^Laboratoire d'Aérologie, Université de Toulouse, CNRS, UPS, Toulouse, France

^9^National Atmospheric Deposition Program (NADP) Champaign, IL, USA

^10^Center for International Climate and Environmental Research – Oslo (CICERO), Oslo, Norway

^11^Asia Center for Air Pollution Research (ACAP), Niigata, Japan

^12^Indian Institute of Tropical Meteorology, Pune, India

^13^Nicholas School of the Environment, Duke University, Durham, NC, USA

^14^Air Resources Laboratory, NOAA, MD, USA

^15^Research Institute for Applied Mechanics, Kyushu University, Fukuoka, Japan

^16^Environment and Climate Change Canada, Toronto, Canada
^17^Chinese Academy of Meteorological Sciences, Key Laboratory for Atmospheric Chemistry, China Meteorological Administration, Beijing, China

*Correspondence to*: Wenche Aas ([waa@nilu.no](mailto:waa@nilu.no)) and Augustin Mortier ([augustinm@met.no](mailto:augustinm@met.no))

**Sites used**

The site selection is largely based on trend analyses done in regional assessments. For EMEP, the same sites were used in Tørseth et al ^1,2^ and Collette et al.^1,2^, as well as the IPCC AR5^3^, though we added the years after 2012. For EANET, NADP, CAPMoN the same precipitation sites are used as in Vet et al.^4^ with an extended time period. The Indian precipitation data used have recently been presented with details on sampling procedures and site descriptions^5^. The IMPROVE data are similar as presented by Hand et al.^6^ though extended to 2015.


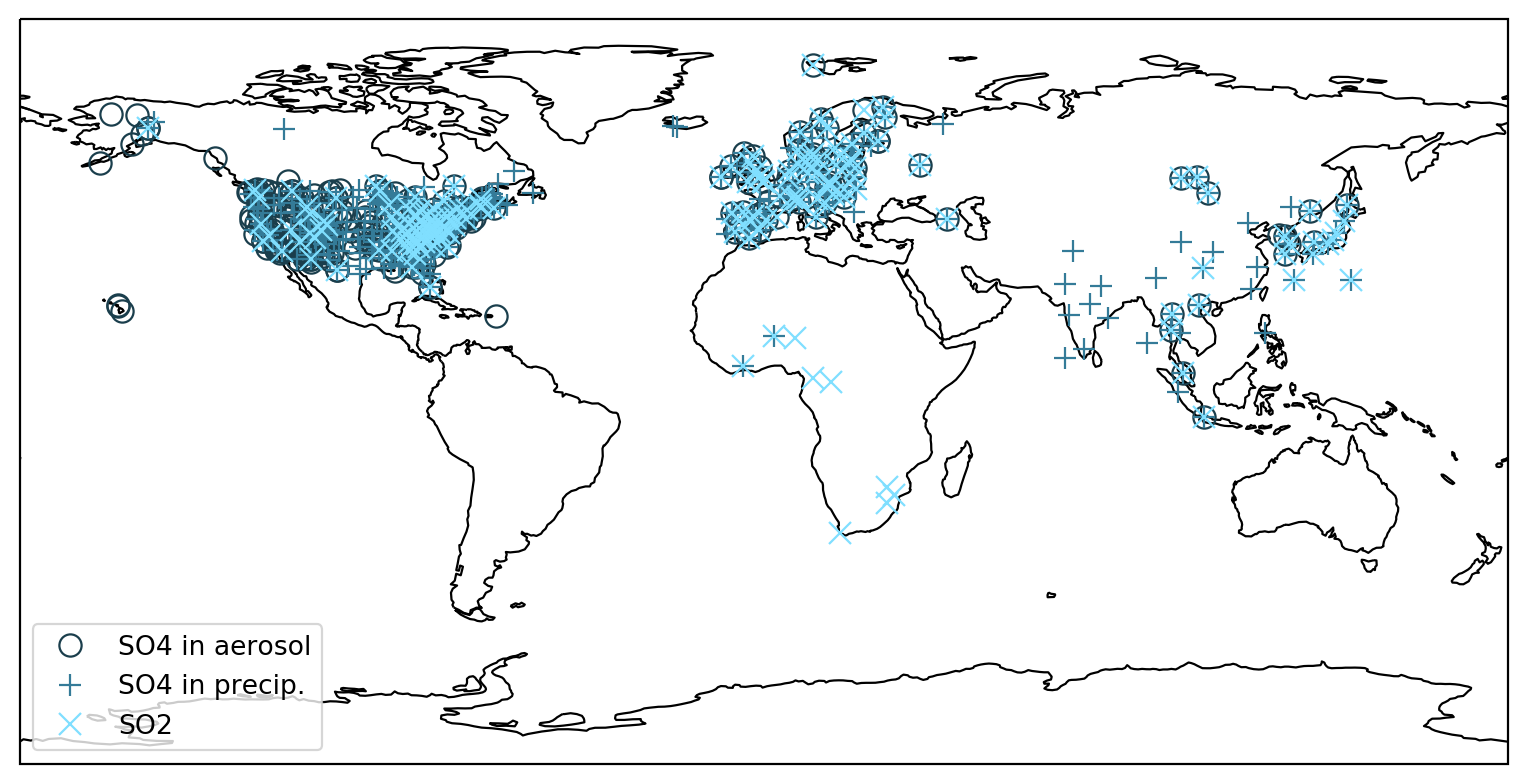


***Figure S1.***  *Location of the sites and measurements used.*

There has been some minor manual selection of sites and correction in the data used in some of the Figures and in the analysis:

- For the 1990-2015 trends (Figures 1, S3 and S4), the 2000 data for many EANET sites have been estimated by using 2001 for several sites which started their program then.
- Some European sites, which lacked measurements for 2015, data for 2014 were used for the 1990-2015 trends (Figures 1, S3 and S4).
- Three French sites were deleted in the statistical calculation for 2000-2010 because they ended in 2007, which in addition showed suspiciously high concentrations affecting the average trend for this period.

**
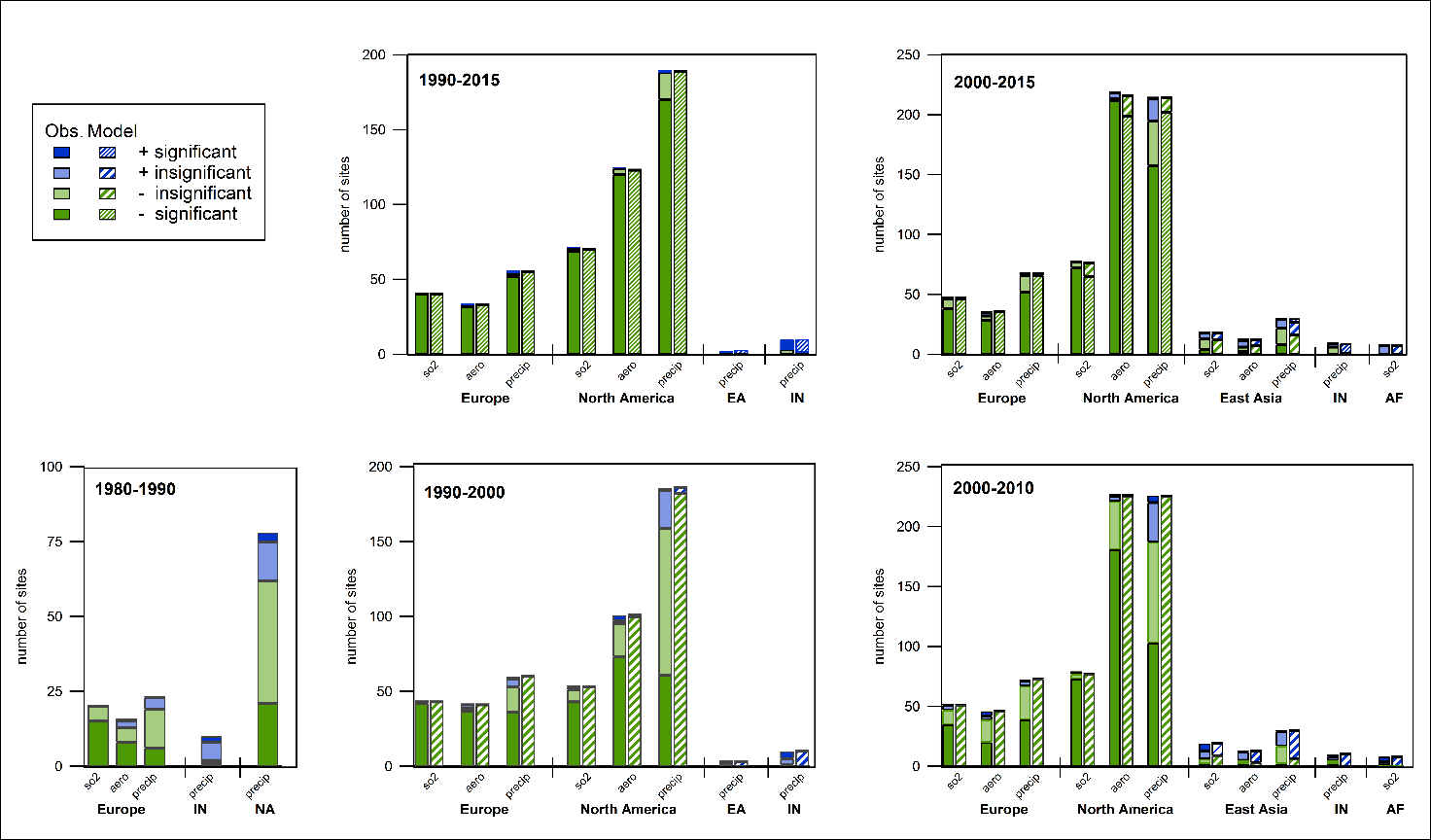
**

***Figure S2.*** *Number of sites with significant and insignificant trends, observed and modeled (EMEP/MSC-W) for different time periods.NA=North America, EA=East Asia, IN=India, AF=Africa. Note that for the ten year periods the model only have three points (five year interval) and cannot detect significance or not.*

***
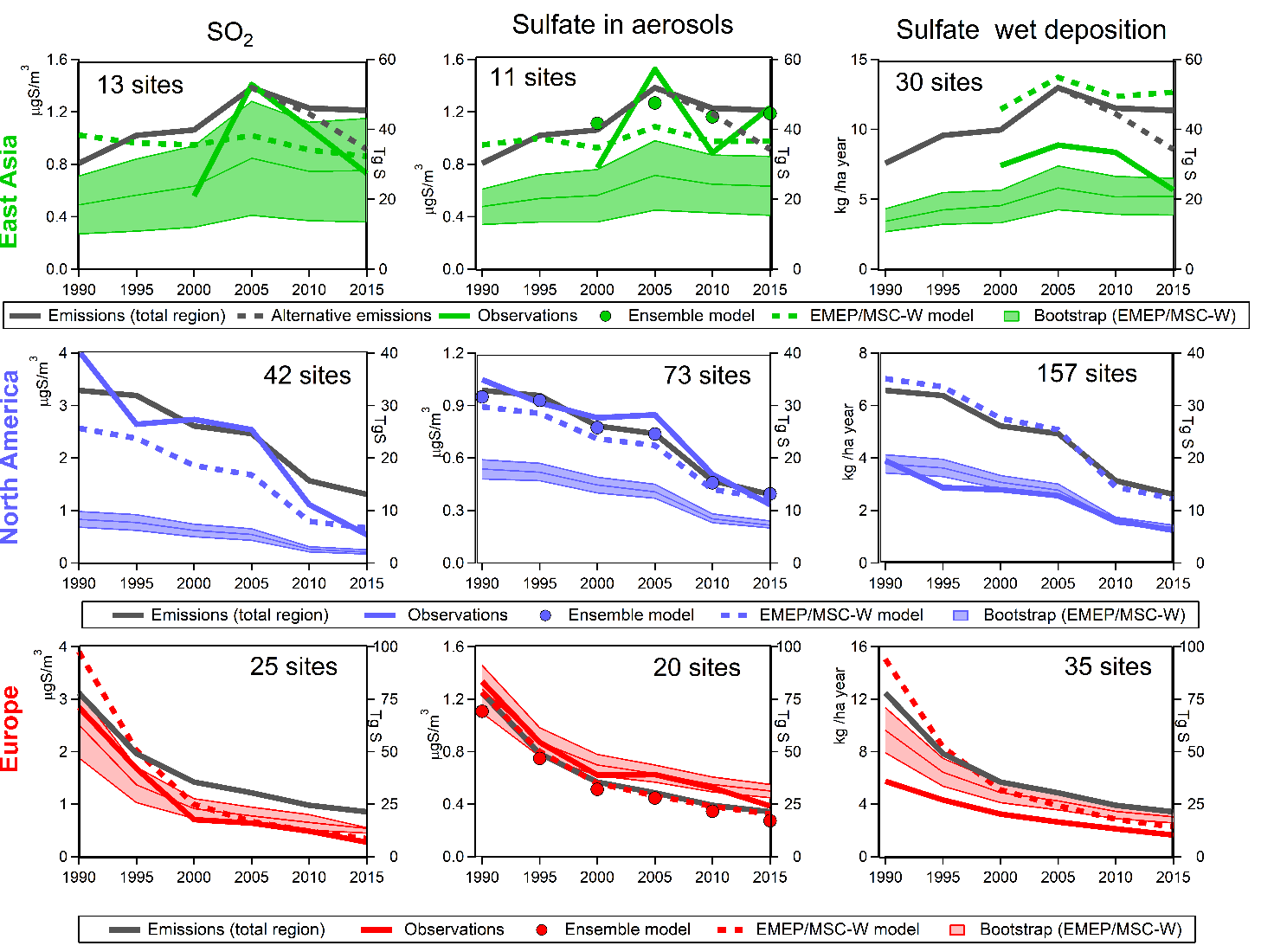
Figure S3.*** *Trends in SO_2_, sulfate in aerosols and in wet deposition in East Asia, North America and Europe from observations and the EMEP/MSC-W model (and Ensemble model for sulfate in aerosols) at the selected number of sites, and the average bootstrap trends and standard deviations from 1000 iterations. Observed and modelled concentrations are given on the y-axis on the left, while emissions on the right. For East Asia an alternative emission development the last ten years are included, based on more recent inventories^7,8^.*

The normalised trends in Figure S4 below have been calculated by setting the 1990 concentration to 0.0 and then adding the relative change for each five year interval. For East Asia the normalised trends were calculated from 2000 for the observations but from 1990 for the emissions and model results. The standard deviation of the bootstrap averages was calculated using the ratio between the bootstrap concentration and standard deviation for the different years.

***
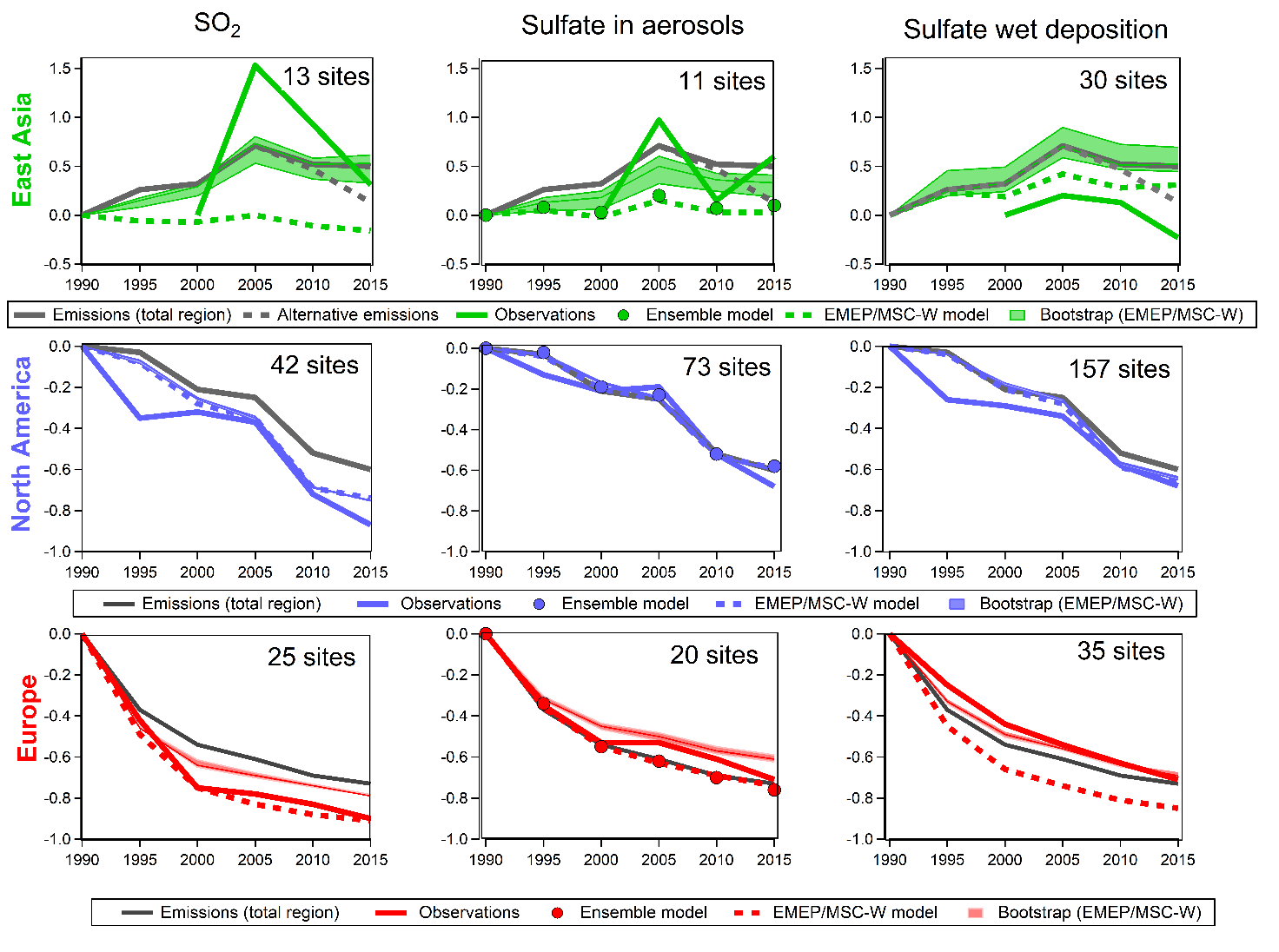
***

***Figure S4.*** *Normalised trends of SO_2_, sulfate in aerosols and in wet deposition in East Asia, North America and Europe in observation and EMEP/MSC-W model (and Ensemble model mean for sulfate in aerosols) at the selected number of sites, and the average bootstrap trends and standard deviations from 1000 iterations. For East Asia an alternative emission development the last ten years are included, based on more recent inventories^7,8^.*

***
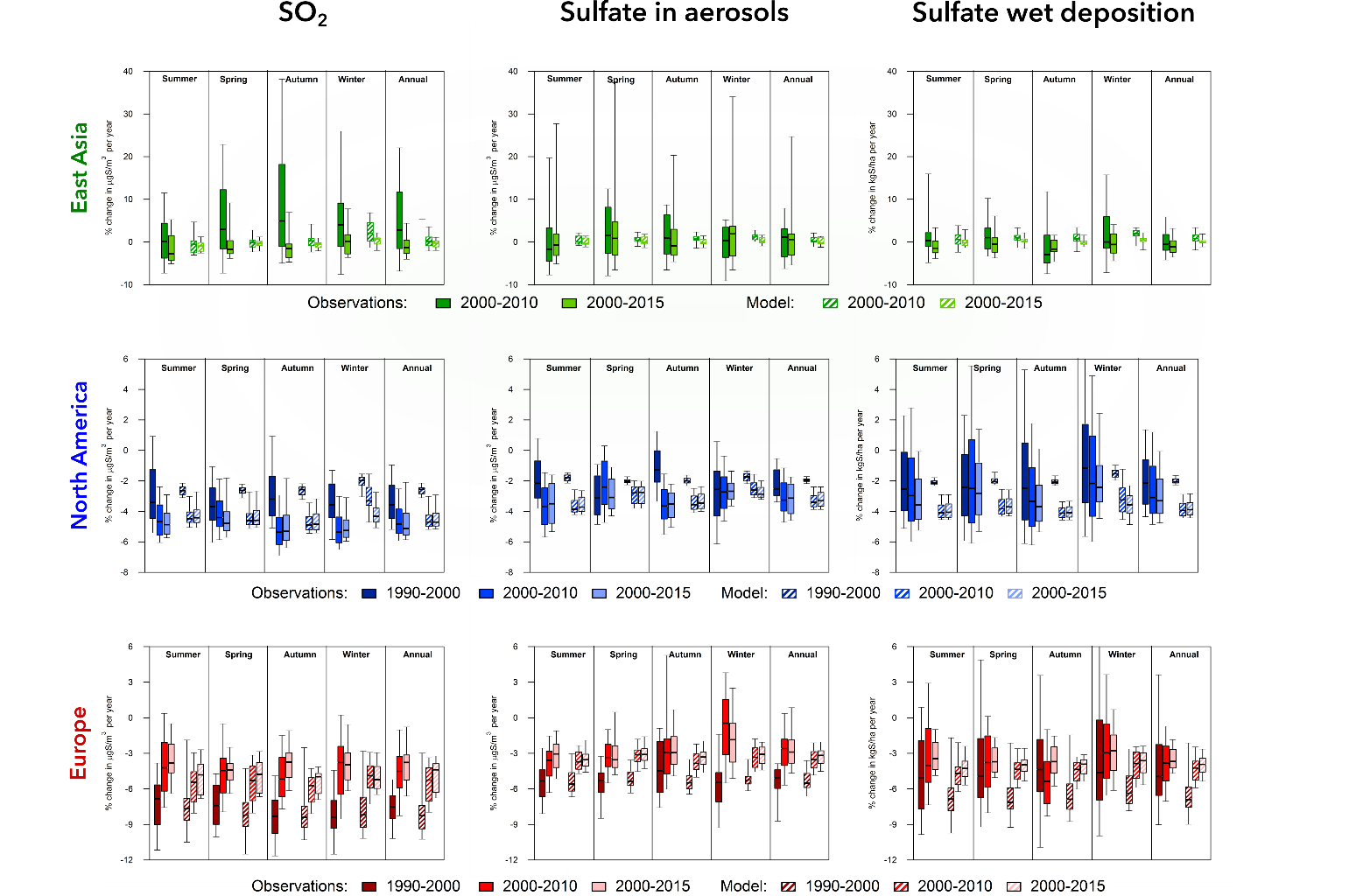
***

***Figure S5.*** *Box blot indicating 10, 25, 50, 75 and 90 percentiles of the seasonal and annual observed and modelled trends (EMEP/MSC-W model for SO_2_ and wet deposition of sulfate; ensemble models for sulfate in aerosols) for different time periods.*

***
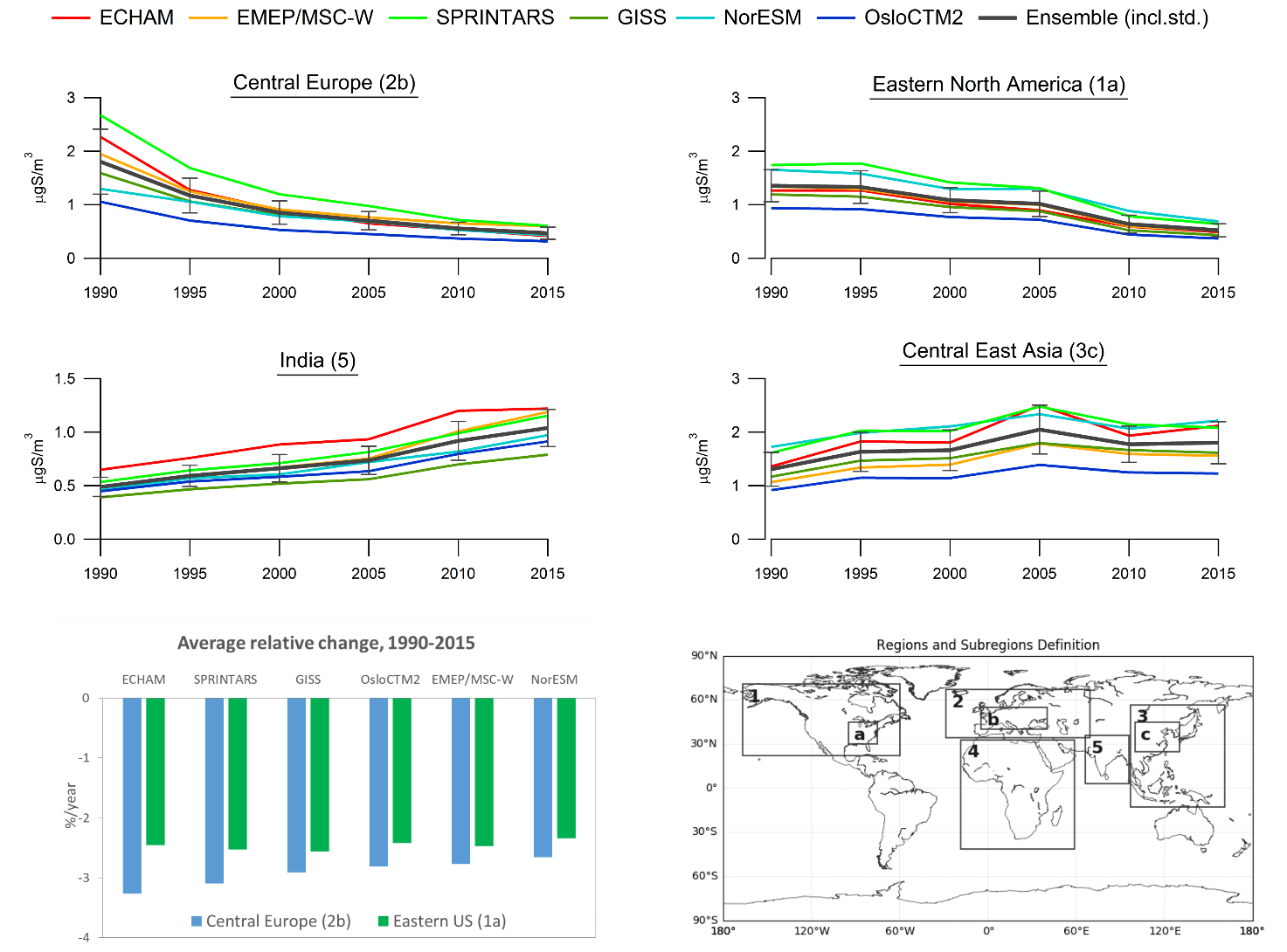
***

***Figure S6.***  *Comparison of the average concentrations and total relative change of modelled sulfate concentrations in aerosols for the period 1990-2015, calculated by the different global models for the different (sub)regions indicated in the bottom right map*

***Table*** *S1. Trends in observed and modelled SO_4_ ^2-^ in aerosols, % change per year*

| **Time period** | **Region** | **Number of stations** | **Average relative trends (STD), % / yr** | | | | | | |
| --- | --- | --- | --- | --- | --- | --- | --- | --- | --- |
|  |  |  | **Obs.** | **ECHAM6** | **EMEP/MSC-W** | **GISS** | **NorESM** | **OsloCTM2** | **SPRINTARS** |
| 1980-1990 | Europe | 16 | -2.56 ( 3.10) |  |  |  |  |  |  |
| 1990-2000 | Europe | 41 | -5.16 ( 2.11) | -5.80 ( 1.26) | -5.80 ( 0.82) | -5.35 ( 1.20) | -3.68 ( 1.63) | -5.02 ( 0.94) | -5.76 ( 1.17) |
| 1990-2000 | North America | 101 | -2.08 ( 1.44) | -2.52 ( 0.62) | -1.91 ( 0.30) | -1.92 ( 0.32) | -1.62 ( 0.70) | -1.71 ( 0.32) | -1.95 ( 0.30) |
| 1990-2015 | Europe | 33 | -2.93 ( 0.69) | -3.52 ( 0.36) | -3.22 ( 0.37) | -2.96 ( 0.55) | -2.47 ( 0.60) | -2.95 ( 0.45) | -3.45 ( 0.53) |
| 1990-2015 | North America | 124 | -2.14 ( 0.67) | -2.40 ( 0.26) | -2.23 ( 0.22) | -2.19 ( 0.36) | -1.95 ( 0.31) | -2.06 ( 0.37) | -2.32 ( 0.23) |
| 2000-2010 | East Asia | 13 | 0.44 ( 4.25) | 0.50 ( 1.43) | 0.40 ( 1.01) | 0.63 ( 0.91) | 0.20 ( 1.16) | 0.34 ( 1.04) | 0.44 ( 1.35) |
| 2000-2010 | Europe | 43 | -2.86 ( 2.20) | -4.34 ( 1.06) | -3.64 ( 1.35) | -3.25 ( 0.96) | -2.69 ( 1.05) | -3.29 ( 0.86) | -3.96 ( 1.07) |
| 2000-2010 | North America | 227 | -3.03 ( 1.72) | -3.70 ( 0.61) | -3.29 ( 0.72) | -3.16 ( 0.83) | -2.75 ( 0.97) | -2.97 ( 0.86) | -3.46 ( 0.63) |
| 2000-2015 | East Asia | 13 | 2.68 ( 9.41) | -0.09 ( 0.86) | 0.02 ( 0.82) | 0.11 ( 0.89) | 0.08 ( 0.97) | 0.02 ( 0.91) | -0.02 ( 0.99) |
| 2000-2015 | Europe | 36 | -2.67 ( 2.03) | -3.58 ( 1.01) | -3.25 ( 1.16) | -3.01 ( 0.79) | -2.79 ( 0.66) | -3.06 ( 0.71) | -3.85 ( 0.79) |
| 2000-2015 | North America | 218 | -3.15 ( 1.30) | -3.43 ( 0.60) | -3.26 ( 0.60) | -3.09 ( 0.79) | -2.86 ( 0.72) | -2.95 ( 0.75) | -3.47 ( 0.52) |

***Table S****2. Trends in observed and modelled SO_4_ ^2-^ in aerosols, change in μgS/m3 per year.*

| **Time period** | **Region** | **Number of stations** | **Average trends in concentration (STD), μgS/m^3^yr** | | | | | | |
| --- | --- | --- | --- | --- | --- | --- | --- | --- | --- |
|  |  |  | **Obs.** | **ECHAM6** | **EMEP/MSC-W** | **GISS** | **NorESM** | **OsloCTM2** | **SPRINTARS** |
| 1980-1990 | Europe | 16 | -0.048 (0.094) |  |  |  |  |  |  |
| 1990-2000 | Europe | 41 | -0.073 (0.052) | -0.082 (0.056) | -0.082 (0.055) | -0.051 (0.037) | -0.036 (0.028) | -0.034 (0.021) | -0.135 (0.110) |
| 1990-2000 | North America | 101 | -0.024 (0.025) | -0.028 (0.014) | -0.020 (0.013) | -0.017 (0.011) | -0.021 (0.014) | -0.011 (0.009) | -0.029 (0.022) |
| 1990-2015 | Europe | 33 | -0.031 (0.015) | -0.042 (0.027) | -0.038 (0.025) | -0.026 (0.018) | -0.022 (0.013) | -0.017 (0.011) | -0.068 (0.046) |
| 1990-2015 | North America | 124 | -0.026 (0.024) | -0.028 (0.016) | -0.022 (0.017) | -0.020 (0.015) | -0.027 (0.019) | -0.015 (0.011) | -0.033 (0.028) |
| 2000-2010 | East Asia | 13 | 0.003 (0.034) | 0.007 (0.018) | 0.005 (0.011) | 0.008 (0.008) | 0.005 (0.020) | 0.003 (0.006) | 0.005 (0.017) |
| 2000-2010 | Europe | 43 | -0.029 (0.041) | -0.034 (0.021) | -0.028 (0.016) | -0.020 (0.013) | -0.020 (0.012) | -0.015 (0.008) | -0.048 (0.028) |
| 2000-2010 | North America | 227 | -0.029 (0.029) | -0.032 (0.018) | -0.025 (0.020) | -0.022 (0.018) | -0.028 (0.020) | -0.016 (0.013) | -0.038 (0.033) |
| 2000-2015 | East Asia | 13 | 0.003 (0.037) | -0.000 (0.010) | 0.001 (0.006) | 0.002 (0.006) | 0.003 (0.012) | 0.002 (0.005) | -0.001 (0.015) |
| 2000-2015 | Europe | 36 | -0.025 (0.028) | -0.030 (0.021) | -0.024 (0.014) | -0.018 (0.011) | -0.020 (0.012) | -0.012 (0.007) | -0.045 (0.025) |
| 2000-2015 | North America | 218 | -0.028 (0.029) | -0.028 (0.017) | -0.021 (0.018) | -0.019 (0.016) | -0.028 (0.021) | -0.014 (0.011) | -0.033 (0.029) |

**References**

1 Colette, A. *et al.* Air pollution trends in the EMEP region between 1990 and 2012. (NILU, Norwegian Institute for Air Research, Kjeller, 2016).

2 Tørseth, K. *et al.* Introduction to the European Monitoring and Evaluation Programme (EMEP) and observed atmospheric composition change during 1972-2009. *Atmospheric Chemistry and Physics* **12**, 5447-5481, doi:10.5194/acp-12-5447-2012 (2012).

3 Hartmann *et al.* Observations: Atmosphere and Surface. (Cambridge, United Kingdom and New York, NY, USA, 2013).

4 Vet, R. *et al.* A global assessment of precipitation chemistry and deposition of sulfur, nitrogen, sea salt, base cations, organic acids, acidity and pH, and phosphorus. *Atmospheric Environment* **93**, 3-100, doi:10.1016/j.atmosenv.2013.10.060 (2014).

5 Bhaskar, V. V. & Rao, P. S. P. Annual and decadal variation in chemical composition of rain water at all the ten GAW stations in India. *Journal of Atmospheric Chemistry* **74**, 23-53, doi:10.1007/s10874-016-9339-3 (2017).

6 Hand, J. L., Schichtel, B. A., Malm, W. C. & Pitchford, M. L. Particulate sulfate ion concentration and SO2emission trends in the United States from the early 1990s through 2010. *Atmos. Chem. Phys.* **12**, 10353-10365, doi:10.5194/acp-12-10353-2012 (2012).

7 Wang, J. *et al.* Particulate matter pollution over China and the effects of control policies. *Science of The Total Environment* **584-585**, 426-447, doi:<https://doi.org/10.1016/j.scitotenv.2017.01.027> (2017).

8 Zheng, B. *et al.* Trends in China's anthropogenic emissions since 2010 as the consequence of clean air actions. *Atmos. Chem. Phys.* **18**, 14095-14111, doi:10.5194/acp-18-14095-2018 (2018).
